# Autonomous control of ventilation through closed-loop adaptive respiratory pacing

**DOI:** 10.1038/s41598-020-78834-w

**Published:** 2020-12-14

**Authors:** Ricardo Siu, James J. Abbas, David D. Fuller, Jefferson Gomes, Sylvie Renaud, Ranu Jung

**Affiliations:** 1 Department of Biomedical Engineering, Florida International University, 10555 W. Flagler St, EC 2602, Miami, FL 33174 USA; 2 School of Biological and Health Systems Engineering, Arizona State University, Tempe, AZ USA; 3 Department of Physical Therapy, Center for Respiratory Research and Rehabilitation, University of Florida, Gainesville, FL USA; 4 Université de Bordeaux, INP Bordeaux, IMS CNRS UMR 5218, 33000 Bordeaux, France

**Keywords:** Biomedical engineering, Computational neuroscience, Respiration, Translational research, Spinal cord

## Abstract

Mechanical ventilation is the standard treatment when volitional breathing is insufficient, but drawbacks include muscle atrophy, alveolar damage, and reduced mobility. Respiratory pacing is an alternative approach using electrical stimulation-induced diaphragm contraction to ventilate the lung. Oxygenation and acid–base homeostasis are maintained by matching ventilation to metabolic needs; however, current pacing technology requires manual tuning and does not respond to dynamic user-specific metabolic demand, thus requiring re-tuning of stimulation parameters as physiological changes occur. Here, we describe respiratory pacing using a closed-loop adaptive controller that can self-adjust in real-time to meet metabolic needs. The controller uses an adaptive Pattern Generator Pattern Shaper (PG/PS) architecture that autonomously generates a desired ventilatory pattern in response to dynamic changes in arterial CO_2_ levels and, based on a learning algorithm, modulates stimulation intensity and respiratory cycle duration to evoke this ventilatory pattern. In vivo experiments in rats with respiratory depression and in those with a paralyzed hemidiaphragm confirmed that the controller can adapt and control ventilation to ameliorate hypoventilation and restore normocapnia regardless of the cause of respiratory dysfunction. This novel closed-loop bioelectronic controller advances the state-of-art in respiratory pacing by demonstrating the ability to automatically personalize stimulation patterns and adapt to achieve adequate ventilation.

## Introduction

Mechanical ventilation is the de-facto approach to maintain proper ventilation when independent breathing is not possible. However, mechanical ventilation poses a risk of alveolar damage^1^ and can lead to diaphragm muscle atrophy. In turn, these factors can delay or prevent subsequent weaning from ventilatory support^1–3^. Direct electrical stimulation of the diaphragm or phrenic nerve has therefore been advanced as alternative means of sustaining breathing after neuromuscular injury or disease. Diaphragm stimulation can elicit functional breaths^4–6^, ameliorate atrophy^7–9^, and reduce the risk of lung damage^10,11^. However, the current technology for controlling a “diaphragm pacer” is not capable of automated, real time adaptation to patient needs. Rather, the pacer is set manually and stimulation parameters remain fixed until further adjustment by a medical practitioner^12^. In health, alveolar ventilation is exquisitely regulated such that arterial levels of carbon dioxide are maintained around a tight “set point” of approximately 40 mmHg. Indeed, even a 1 mmHg change in arterial CO_2_ can cause a significant change in breathing. This tight matching of breathing with CO_2_ delivery is required to maintain acid/base homeostasis and is a fundamental aspect of ventilatory control in humans. Therefore, the objective of the current work was to develop a closed-loop diaphragm-pacing controller that mimics the endogenous biological controller by responding to changes in metabolic demand with appropriate changes in ventilation, but without the need for manual manipulation of pacing parameters.

**Figure 1:**
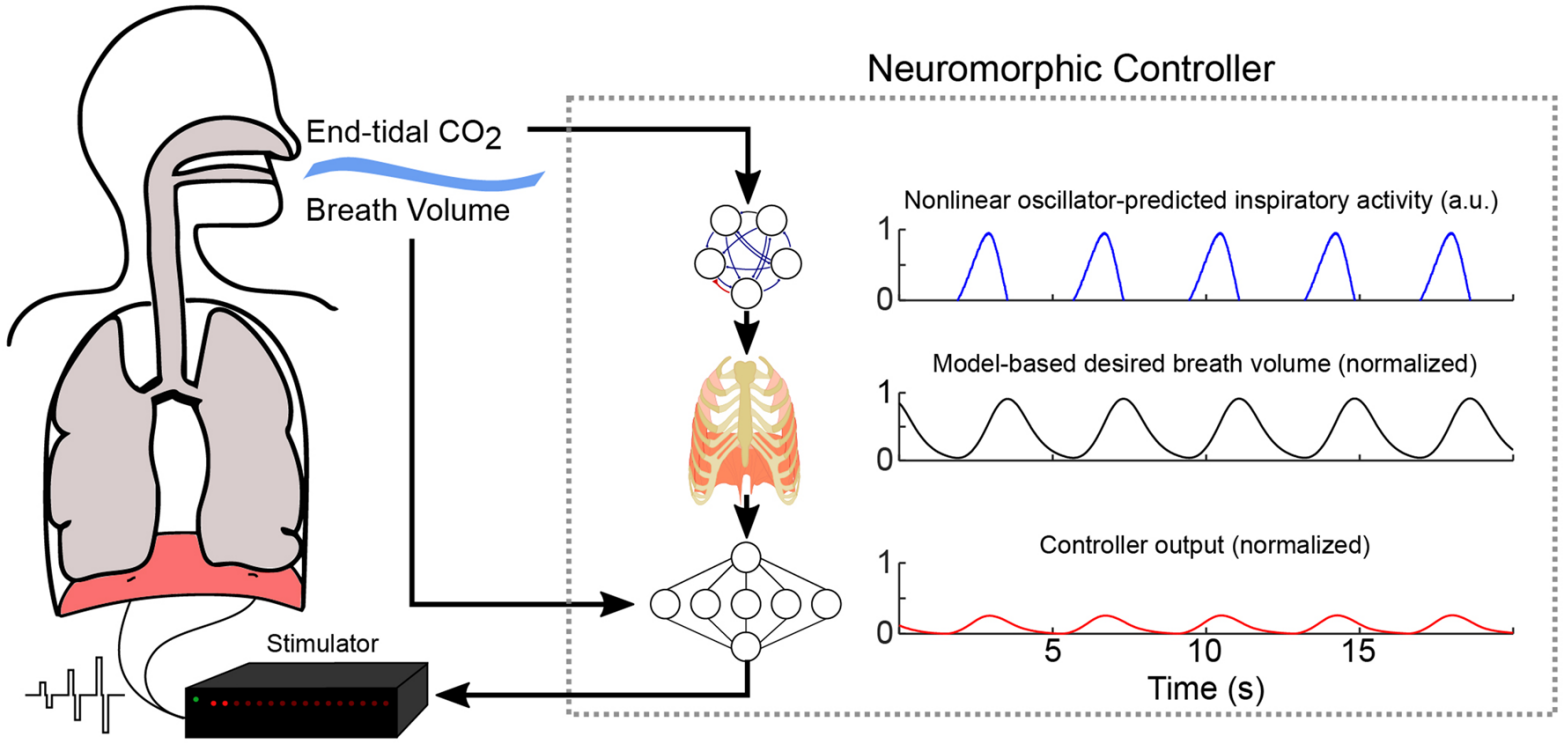
General concept for the adaptive neuromorphic closed-loop control system for respiratory pacing. The adaptive controller uses measurements of end-tidal CO_2_ to predict, through a model-based approach, an adequate ventilatory response. The controller then uses the measured breath volume to autonomously tune pacing parameters such that the ventilatory pattern evoked by diaphragmatic stimulation matches the “desired” ventilatory pattern. This two-stage closed-loop approach allows for a respiratory pacing system that can adapt, on a breath-by-breath basis, to continuously adjust pacing to evoke an adequate ventilatory response.

A neuromorphic closed-loop adaptive controller was developed and first evaluated in silico and then in vivo using two animal models of hypoventilation. The controller automatically adapts to changes in expired end-tidal CO_2_ levels (etCO_2_) and prescribes a ventilatory pattern on a breath-by-breath basis. The etCO_2_ is easily measured and may be used as a practical, albeit not perfect, alternative for direct arterial CO_2_ measurement^13,14^. The controller produces, in real-time, the prescribed ventilatory pattern by modulating diaphragm muscle stimulation. The approach enables generation of the desired dynamic volume profile within a given breath, thereby preventing respiratory acidosis or alkalosis if metabolic (and thus respiratory) demand changes. This biologically-inspired adaptive closed-loop respiratory pacing control scheme (Fig. 1) is the first ever for management of arterial CO_2_ during respiratory pacing. Closed-loop control of etCO_2,_ and by extension arterial CO_2_, in people with ventilatory impairments would not only alleviate concerns about inadequate ventilation during low-intensity activities but may also allow ambulatory and/or partially ambulatory patients to lead a more active lifestyle without risk of hypoventilation, thereby leading to improvements in health and quality of life.

## Materials and methods

### Surgical procedures

Animal use was approved by the Institutional Animal Care and Use Committee of Florida International University. All experiments were performed in accordance with relevant guidelines and regulations. Studies were completed in anesthetized, spontaneously breathing, adult, male Sprague–Dawley rats with an intact spinal cord (Group 1, *n* = 7, 411 ± 91 g., 3.6 ± 0.9 months) or with a left cervical (C2) spinal cord hemisection (Group 2, *n* = 6, 390 ± 80 g., 3.4 ± 0.8 months)^15–19^. Rats were anesthetized with urethane (1.5 g/kg, s.c.) and supplemental isoflurane (0.2–1.5% in 100% O_2_). Body temperature and plane of anesthesia were monitored throughout the experiment. After tracheostomy, airflow was measured using a pneumotachometer (PTM Type HSE-73-0980, Harvard Apparatus, Holliston, MA) and integrated (0.2 s time constant; PI-1000, CWE Inc, Ardmore, PA) to provide breath volume. End-tidal CO_2_ was monitored (CapStar-100, CWE Inc., Ardmore, PA) as a proxy for the partial pressure of arterial CO_2_ (PaCO_2_). Hemi-diaphragmatic electromyograms ipsilateral to the spinal hemisection were used to confirm functional hemiparesis^20^. At experiment termination, the spinal cord tissue was harvested and later histologically assessed for hemisection verification^18^.

### Diaphragm stimulation

Intramuscularly implanted single-stranded, stainless steel electrodes (SS-304, 44 AWG, AM-Systems, Carlsborg, PA) were used to stimulate each hemidiaphragm^18^. A stimulator (FNS-16, CWE Inc., Ardmore PA) delivered biphasic cathodic-first current pulses (200 µs/phase, 80 µs inter-phase interval at 72 Hz) at a variable current amplitude determined by the controller at a maximum of four times the twitch threshold.

### Experimental protocol

The goal was to assess whether the PG/PS controller could autonomously control breath volume and respiratory rate to restore normocapnia. Hypoventilation, with associated hypercapnia, was induced by either delivery of additional isoflurane, a known respiratory depressant^21,22^, or via spinal cord injury (SCI). An elevation of etCO_2_ served as a marker of hypoventilation. In the respiratory depressed group, once an etCO_2_ value of 50 mmHg or more was reached, the pacing trial was initiated. In the SCI group, baseline ventilatory recordings were collected after electrode implantation but prior to SCI. A period of at least 30-min served to stabilize the immediate effects of the injury. After the stabilization period and when etCO_2_ exceeded 50 mmHg, the pacing trial was initiated.

Trials consisted of 60 s of spontaneous breathing after which the PG/PS was enabled; pacing was maintained for at least 900 s without intervention. On cessation of pacing, a 30 min rest period ensued. If etCO_2_ exceeded 50 mmHg during rest, mechanical ventilatory support was provided to reduce etCO_2_ below 50 mmHg. In all trials, the desired ventilatory pattern was determined by the PG module of the controller based on etCO_2_ feedback and animal body weight as described above. PG/PS controller-based pacing was considered successful if etCO_2_ was reduced to within a normocapnic range (36 ± 7 mmHg) during stimulation.

### In vivo performance measures

The performance of the PG/PS controller was evaluated by measuring ventilation. An “adequate ventilation” was determined by whether or not etCO_2_ values returned towards normocapnic levels and if normocapnia was maintained throughout the trial. The ability of the PG/PS controller to maintain the desired ventilatory pattern was assessed by measuring the inspiratory root mean square error (iRMSE), which provides a measure of the error between the elicited volume and the desired volume profile^18^.

The first 20 cycles of iRMSE after entrainment and the last 20 cycles of the trial were compared to assess whether controller performance declined over time. The controller’s ability to achieve normocapnia was calculated as the average decrease in etCO_2_ from the 20 breath cycles obtained after pacing was initiated and entrainment had occurred to the last 20 cycles of the 1000 cycle trial.

### Statistical analysis

A general linear mixed model^23^ was used to assess the effect of PG/PS controlled pacing on etCO_2_. Fixed effects consisted of trial number and condition (prior to pacing and after pacing) and random intercept effects of both trial number and measurement occasion (last 20 breath measurements within each trial). All descriptive statistics are given in the form of mean ± standard deviation. The generalized linear mixed model was performed using SAS 9.4 (SAS Institute Inc., Cary, NC).

### Adaptive pattern generator (PG)/adaptive pattern shaper (PS) controller design

**Figure 2:**
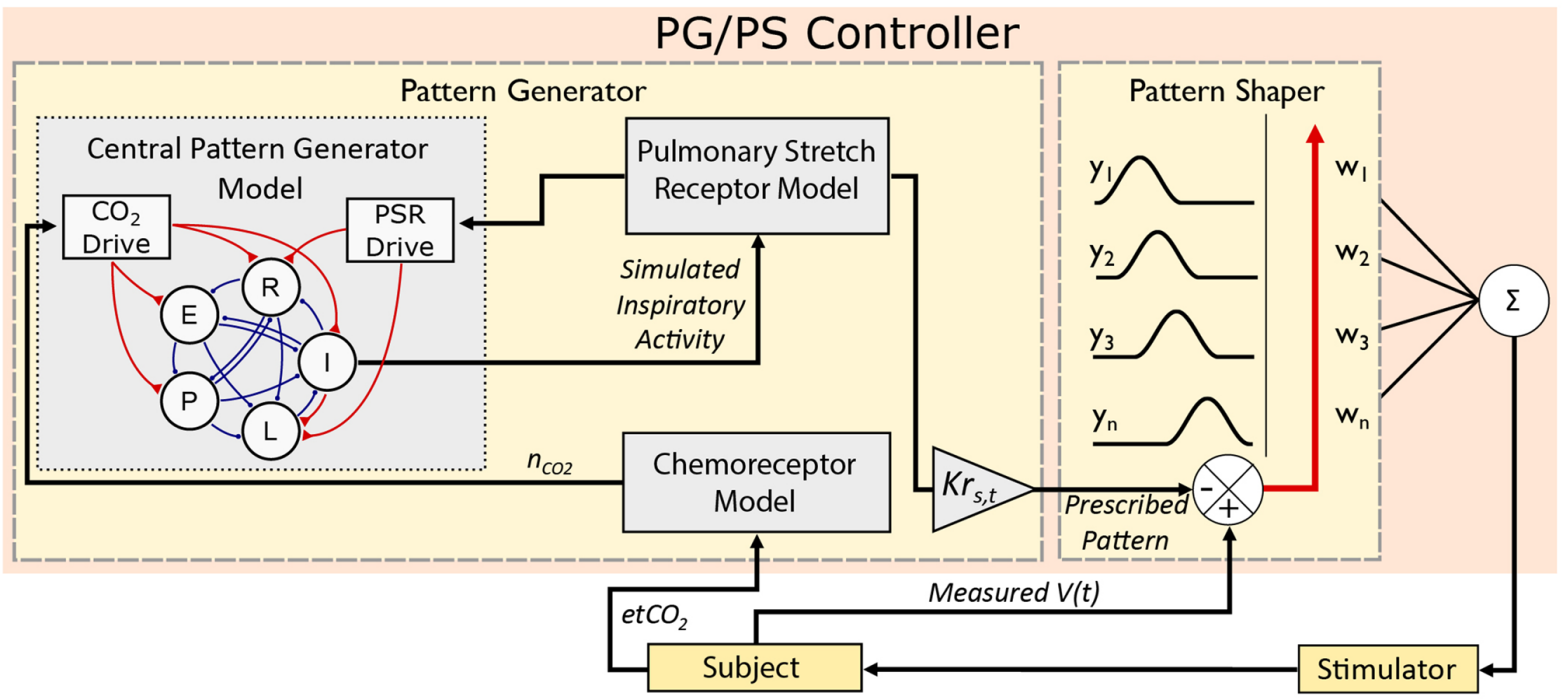
Pattern generator/pattern shaper adaptive controller block diagram. A Pattern Generator (PG) is used to prescribe a ventilatory pattern and a Pattern Shaper (PS) is used to determine the stimulation required for pacing of the diaphragm to attain the prescribed pattern. The PG integrates a computational model of the respiratory central pattern generator (rCPG)^19^, a pulmonary stretch receptor (PSR) model, and a CO_2_ chemoreceptor model to generate a ventilatory response, which is scaled in amplitude by a factor *Kr*_*s*_ and in time by a nominal factor *Kr*_*t*_ to prescribe a breath volume profile to be elicited. The PS derives a volume-based error measure used to continuously modulate network weights w_n_, which ultimately help define the amplitude of the stimulation delivered to the diaphragm. Changes in PaCO_2_ due to changes in ventilation are reflected in changes in etCO_2_. The chemoreceptor model converts etCO_2_ to neural CO_2_ drive *nCO*_*2*_ which modulates activity within the rCPG, generating a new inspiratory pattern and closing the control loop. Breath volume at time t, *V(t)*; partial pressure of arterial CO_2_, *PaCO*_*2*_; end-tidal CO_2_, *etCO*_*2*_; Neural CO_2_ drive from chemoreceptor, *nCO*_*2*_; *Kr*_*s*_ amplitude scaling factor; *Kr*_*t*_ duration scaling factor.

The neuromorphic controller is inspired by a Pattern Generator (PG)/Pattern Shaper (PS) scheme previously implemented for lower limb control^24,25^. Here, a mathematical model of the respiratory central pattern generator (rCPG) serves as the basis for respiratory rhythmogenesis (Fig. 2). An adaptive PG module integrates the rCPG with a chemoreceptor model and a pulmonary stretch receptor model to prescribe an appropriate ventilatory pattern in response to changes in PaCO_2_ (in silico) or etCO_2_ (in vivo) on a breath-by-breath basis. The PG module is coupled to an adaptive pattern shaper (PS) module which leverages an adaptive neural network to determine stimulation parameters for diaphragmatic pacing to evoke the PG-prescribed ventilatory pattern^18^. Supplementary material provides detailed model descriptions and controller and model parameter values.

Specifically, the desired breath volume profile is generated automatically by the PG module. The PG module utilizes a triphasic oscillatory network of five interconnected neuronal pools of respiratory neurons to mimic the behavior of the respiratory CPG^26^. For every breath, it uses model-based chemoreceptor drive and model-based pulmonary stretch receptor feedback drive to determine an appropriate inspiratory duration for the next breath. The chemoreceptor drive to the PG is a bounded linear function of PaCO_2_ in silico (35–45 mmHg) and etCO_2_ (30–45 mmHg) in vivo (see Supplementary material for computational model). These bounds limit the maximum breath amplitude produced by the PG module, thus guarding against volutrauma. To minimize unwanted ventilatory responses to spurious changes in PaCO_2_ in silico or etCO_2_ in vivo, an exponential moving average (EMA) of the peak PaCO_2_/etCO_2_ was utilized (time constant = 8 s). The output from the pulmonary stretch receptor model was used as an additional drive for rhythmogenesis^26^, resulting in a ventilatory response to PaCO_2_/etCO_2_ that matched that observed in mammals. The Supplementary Material provides a detailed description of the PG module and the pulmonary stretch receptor and chemoreceptor computational models.

The output of the inspiratory pool of the rCPG was half-wave rectified and processed through the pulmonary stretch receptor model. The pulmonary stretch receptor output was then scaled in amplitude to match the tidal volume expected for the weight of each rat^27^. This output was also scaled in time to match endogenous respiratory rates during eupneic^27,28^ and hypercapnic^28,29^ conditions. This ventilatory pattern was obtained on a breath-by-breath basis, serving as the prescribed trajectory for the PS module to follow. In the experimental studies, if etCO_2_ information was unavailable (e.g. first breath of pacing), the controller worked under the assumption that the etCO_2_ input was 36 mmHg.

The PS module, a neural network with 72 neurons time-shifted every 0.014 s, aims to determine adequate stimulation parameters to elicit a specified breath volume profile^18^. Unlike previously, this prescribed profile now also varies in cycle duration. Hence, to account for changes in the prescribed breath cycle duration, neurons in the network are silenced or reactivated at the start of every breath to modulate the pacing cycle duration. In this manner, the PS is able to work in concert with the PG module to evoke the prescribed ventilatory pattern.

The PG/PS controller was programmed and implemented in LabVIEW (National Instruments, Austin, TX). In animal studies, the controller output to the stimulator was scaled such that the maximum stimulator output was four times the twitch threshold^18^.

The controller was assessed in silico to test functionality prior to in vivo studies. It’s ability to respond to changing metabolic conditions was assessed in vivo in two animal models of hypoventilation-induced hypercapnia.

### Computational testbed for controller development

**Figure 3:**
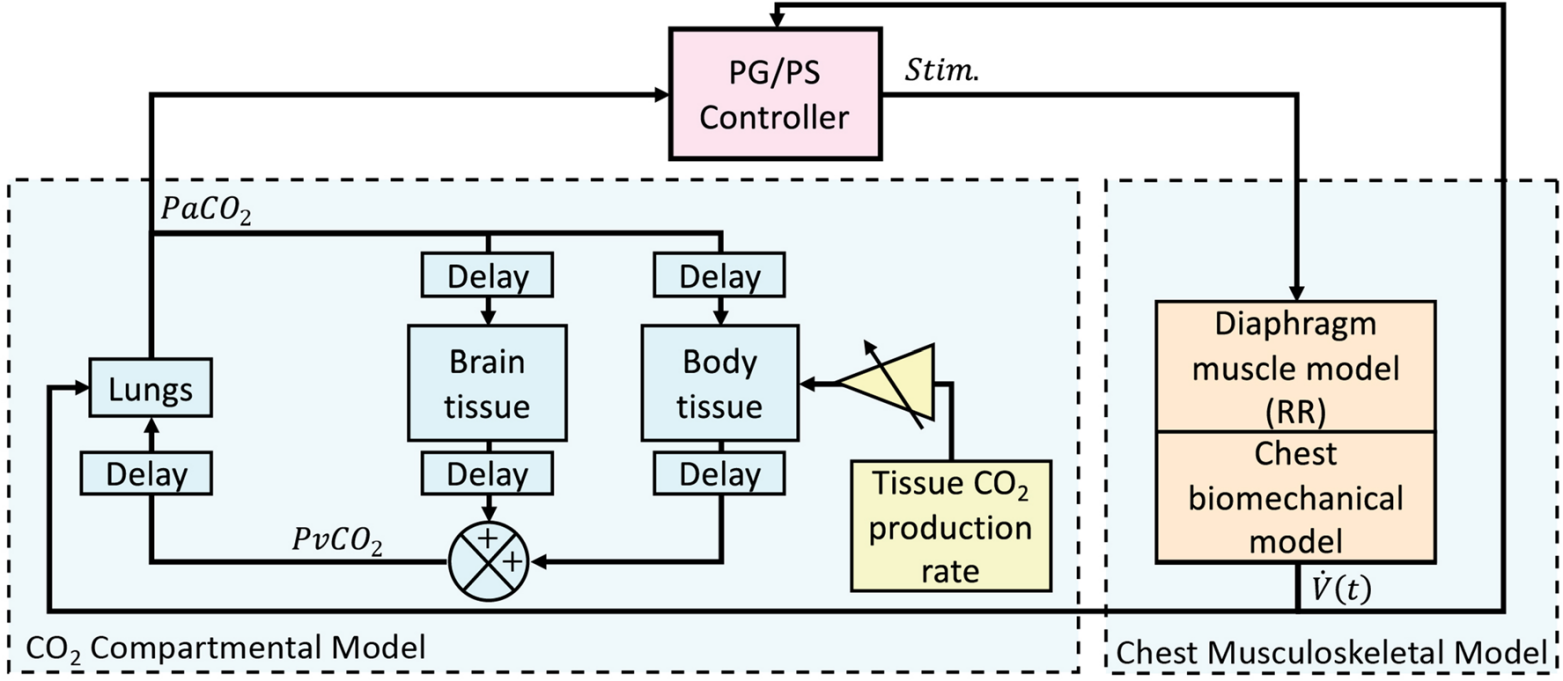
Block diagram of the computational testbed used to assess the PG/PS controller in silico. A chest musculoskeletal model with reverse recruitment (RR) dynamics and a CO_2_ compartmental model were linked with the PG/PS controller. The ventilation, *V̇(t)*, elicited by the chest musculoskeletal model drives changes in PaCO_2_ in the compartmental model of CO_2_ dynamics. PaCO_2_ is sampled by the controller which then drives force generated by the muscle model. Changes in the gain of CO_2_ production rate within the tissue (yellow) were used to simulate changes in metabolic demand. The eventual change in PaCO_2_ was used to assess the controller’s ability to maintain normocapnia despite changes in metabolic demand that may result from increased exercise or other changes in metabolic activity. Model parameters and values were obtained from literature. Breath volume at time t, *V(t)*; partial pressure of arterial CO_2_, *PaCO*_*2*_; partial pressure of venous CO_2_, *PvCO*_*2*_.

The PG/PS controller was developed and validated computationally prior to in vivo assessment. A comprehensive computational model containing biomechanical, muscular, and CO_2_ dynamics was developed based on a previously published model for rat musculoskeletal dynamics^18^. A computational model for CO_2_ generation in humans^30–32^ was adapted and scaled to match rat normative values found in literature^27,29^ and integrated with the biomechanical model. The organization of these models and their integration with the adaptive PG/PS controller is illustrated in Fig. 3. A list of constants and variables used for these models can be found in Supplementary Table S1 online.

The model describing CO_2_ dynamics used is that of a CO_2_ compartmental model containing a general body tissue compartment, a brain compartment, and a lung compartment with appropriate CO_2_ transport delays^29^. The body tissue and brain compartments produce CO_2_ based on compartment volumes and a rate of CO_2_ production as described below for the body tissue compartment.

1 $$ V_{T} \frac{{dC_{T} }}{dt} = \dot{M}_{T} + \dot{Q}_{T} \left( {C_{a} + C_{T} } \right) $$

The concentration of CO_2_ of the compartment, *C*_*T*_, is given by volume of the compartment, *V*_*T*_, metabolic rate of the compartment, *Ṁ*_*T*_, given as CO_2_ production rate, perfusion through the tissue compartment, ${\dot{Q}}_{T}$, and arterial CO_2_ concentration from the arterioles to the compartment tissue, *C*_*a*_. An identical equation is used to determine CO_2_ concentration derived from the brain tissue compartment by replacing volume, metabolic rate, perfusion rate with the respective values for the brain tissue. The total venous CO_2_ concentration, *C*_*V*_, is given by the concentration of venous CO_2_ coming from the brain and body tissue compartments, *C*_*vB*_ and *C*_*vT*_ respectively, and their respective perfusion rates.

2 $$ \frac{{dC_{v} }}{dt} = \dot{Q}_{B} C_{vB} + \dot{Q}_{T} C_{vT} $$

The concentration of CO_2_ within the alveoli is given using the lung compartment equation defined as

3 $$ \frac{{dVC_{a} }}{dt} = \left\{ {\begin{array}{ll} {\dot{Q}\left( {C_{V} - C_{a} } \right) + \dot{V}C_{in} } & {\dot{V} \ge 0} \\ {\dot{Q}\left( {C_{V} - C_{a} } \right) + \dot{V}C_{a} } & {\dot{V} < 0} \\ \end{array} } \right. $$

where arterial CO_2_ concentration is given by lung volume, *V*, venous CO_2_ concentration, total perfusion, $\dot{Q}$, inspired CO_2_ concentration, *C*_*in*_ and ventilation, $\dot{V}$.

### Computational studies for PG/PS in silico characterization and assessment

The time constant of the EMA was selected based on simulations to assess the controller’s response to two different physiological scenarios: a sudden increase in metabolic demand and apnea. The sudden increase in metabolic demand was modelled by increasing CO_2_ production in the tissue by 50% at 180 s after pacing initiation. Apnea was simulated by setting diaphragm activation to zero for one cycle 300 s after pacing initiation. Time constants of 1, 2, 4, 6, 8, 10, 15, 20, 25, and 30 s were used for the EMA. The controller’s response was assessed via the root mean square error (RMSE) between the measured PaCO_2_ response and an idealized CO_2_ response from 5 s prior to the increase in CO_2_ rate production to 60 s after the apneic event. The standard deviation in RMSE was used to characterize stability after perturbation while the maximum RMSE was used to assess magnitude of the overshoot caused by the controller after the perturbation^33^.

To assess the controller’s ability to control ventilation reliably in response to changes in PaCO_2_ in a closed-loop manner, simulations were performed with varying rates of CO_2_ production in the tissue. The value for CO_2_ production rate was modified to be 10–200% of baseline CO_2_ production after 180 s to simulate changes in respiratory demand due to metabolic activity. The ability to achieve normocapnia by the end of the trial was used to assess the controller’s performance. The adaptive PG/PS controller was compared to a version of the controller with a fixed PG pattern that did not have the ability to directly respond to PaCO_2_, but had an adaptive PS that attempted to match the breath volume profile.

### In silico performance measures

In computational trials, the PG/PS controller’s ability to determine a prescribed ventilatory output was assessed by observing the change in prescribed tidal volume and prescribed breath duration with respect to the model’s PaCO_2_ output and comparing this trend to the expected ventilatory response to hypercapnia found in literature.

## Results

### Computational studies confirm ability to respond to metabolic demand

**Figure 4:**
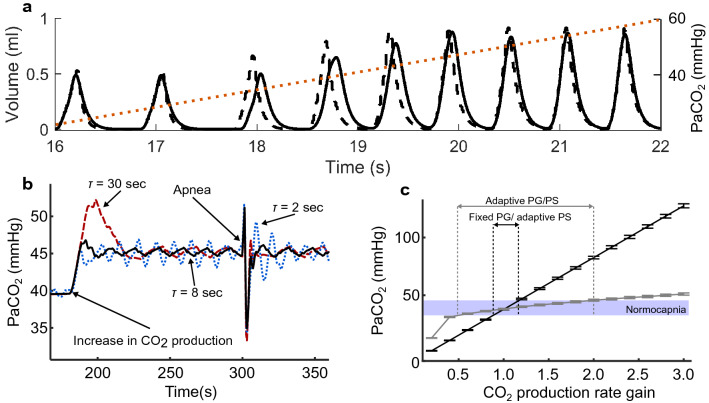
Adaptive PG/PS controller performance assessed in a computational testbed. (**a**) Adaptive PG, adaptive PS response to a ramping increase in PaCO_2_ values. The PG module generates a prescribed ventilatory profile (dashed) in response to a controlled increase in PaCO_2_ levels (dotted). The PS module responds by adapting stimulation to elicit a breath volume (solid) matching that of the pattern dictated by the PG. (**b**) Evaluation of the adaptive PG/PS response to PaCO_2_ under chemo-transducer time constants of 2 s (dotted blue), 8 s (solid black), and 30 s (dashed red). A time constant of 8 s provided the best stability and robustness to perturbations. (**c**) Comparison between a fixed PG/adaptive PS (solid black) and an adaptive PG/PS controller’s (solid grey) ability to maintain normocapnia. The adaptive PG/PS can maintain normocapnia across a wider range of the baseline CO_2_ production rate than the fixed PG/adaptive PS.

Computational studies showed that the controller could compute and produce a ventilatory pattern capable of restoring normocapnia when provided with a wide range of PaCO_2_ values. Figure 4a shows that the controller responds to an increase in PaCO_2_ by increasing tidal volume and decreasing cycle period.

Figure 4b demonstrates the controller’s response to sudden changes in PaCO_2_ under time constant values of 2, 8, and 30 s. After a 50% increase in CO_2_ production, low values of *τ* led to a fast response but unstable oscillatory behavior long after the perturbation. Larger values achieved stability but caused a significant overshoot in PaCO_2_ after apnea. Overall, a *τ* of 8 s led to a small overshoot with minimal underdamping as represented by the lowest PaCO_2_ root mean square error (RMSE) among all other *τ* values.

Comparison between a version of the controller with a fixed-PG (non-responsive to changes in PaCO_2_) and a version with an adaptive PG (closed-loop PaCO_2_ control) demonstrates that the fixed-PG controller was able to maintain normocapnia within 89–115% of the baseline CO_2_ production rate, while the adaptive PG controller was able to achieve normocapnia within a larger range of 52–200% of the baseline CO_2_ production rate, as seen in Fig. 4c.

### Restoring normocapnia after anesthesia-induced hypoventilation

**Figure 5:**
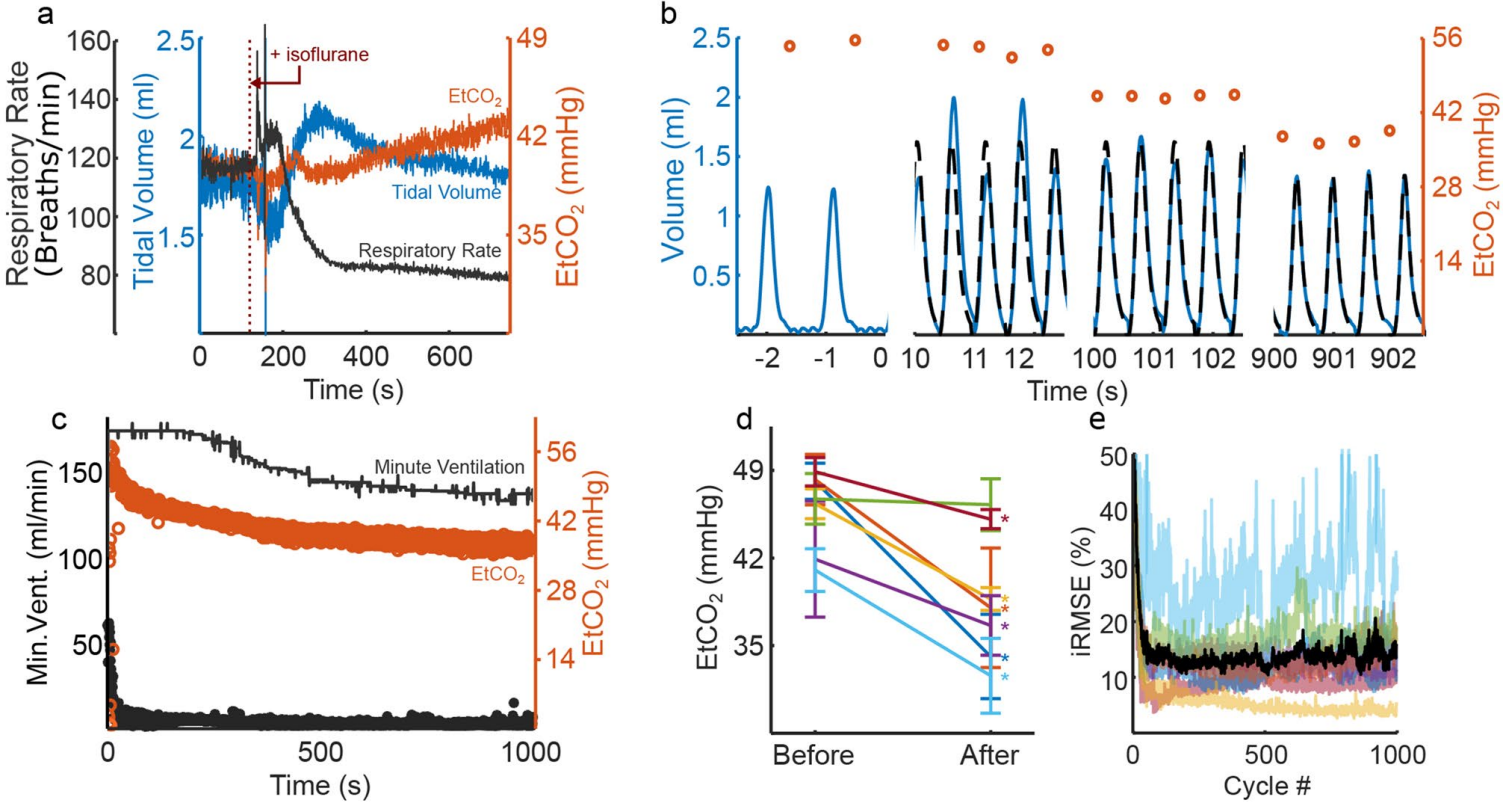
Adaptive PG/PS controller use in vivo after anesthesia-induced hypoventilation. (**a**) Supplemental isoflurane (+ 1.5%, 100% O_2_) caused hypoventilation mainly via a decrease in respiratory rate (black), which led to an increase in etCO_2_ (orange). (**b**) Ventilatory pattern prior to controller initiation (0 s), following ventilatory entrainment, 100 s, and 900 s after controller initiation. The adaptive PG/PS was able to respond to the elevated peak etCO_2_ (orange, circles) by eliciting a hyperventilatory pattern (black, dashed line) and adapting stimulation to match the measured volume (blue, solid line) to the desired pattern. After 900 s, etCO_2_ is within acceptable levels and thus the ventilatory pattern elicited shows a decreased tidal volume and respiratory rate. (**c**) Adaptive PG/PS controller response throughout a 1000 s trial. The adaptive PG/PS controller responds to an elevated etCO_2_ (orange, empty circle) by dictating a ventilatory pattern with high minute ventilation (grey). This in turn causes a decrease in etCO_2_ towards normocapnia. Throughout the trial, iRMSE (black, solid circles) remains low (< 10%) showing that the PS controller is able to match the pattern dictated by the PG. (**d**) The adaptive PG/PS controller was able to significantly decrease etCO_2_ and (**e**) maintain low iRMSE after anesthesia-induced hypoventilation (*n* = 6); One trial (cyan, rat #6) showed elevated iRMSE due to loss of entrainment. A low tidal volume resulted from low etCO_2_, decreasing likelihood of entrainment. Black iRMSE line denotes average across all animals.

Adaptive closed-loop pacing alleviated hypercapnia in animals with anesthesia-induced respiratory depression. Anesthesia caused hypoventilation as shown by the increased etCO_2_ in Fig. 5a. Figure 5b shows how the adaptive closed-loop controller automatically altered respiratory rate and breath volume in response to hypercapnia and adapted stimulation to account for dynamic changes in the desired ventilatory pattern throughout the trial. In response to elevated etCO_2_, initially the controller elicited high minute ventilation, which led to a drop in etCO_2_. As the trial progressed, the controller adapted and responded to a decrease in etCO_2_ with reduced minute ventilation, eventually leading to and maintaining normocapnia (Fig. 5c). The adaptive PG/PS controller maintained a low iRMSE throughout the trial, confirming that the PS module was able to evoke the desired ventilatory pattern set by the PG module.

A summary of the effects of pacing on etCO_2_ and iRMSE across all animals is presented in Fig. 5d,e, respectively. Overall, etCO_2_ decreased by 7.8 ± 2.6 mmHg (*p* = 0.0031) from when the controller was enabled to the end of the trial. An average iRMSE of 13.64 ± 1.44% after entrainment also shows that the PS module adapted. Overall, the PG/PS controller produced an adequate ventilatory pattern to restore regular ventilatory capabilities after reduced ventilatory drive due to central respiratory depression.

### Restoring normocapnia after trauma-induced hypoventilation

**Figure 6:**
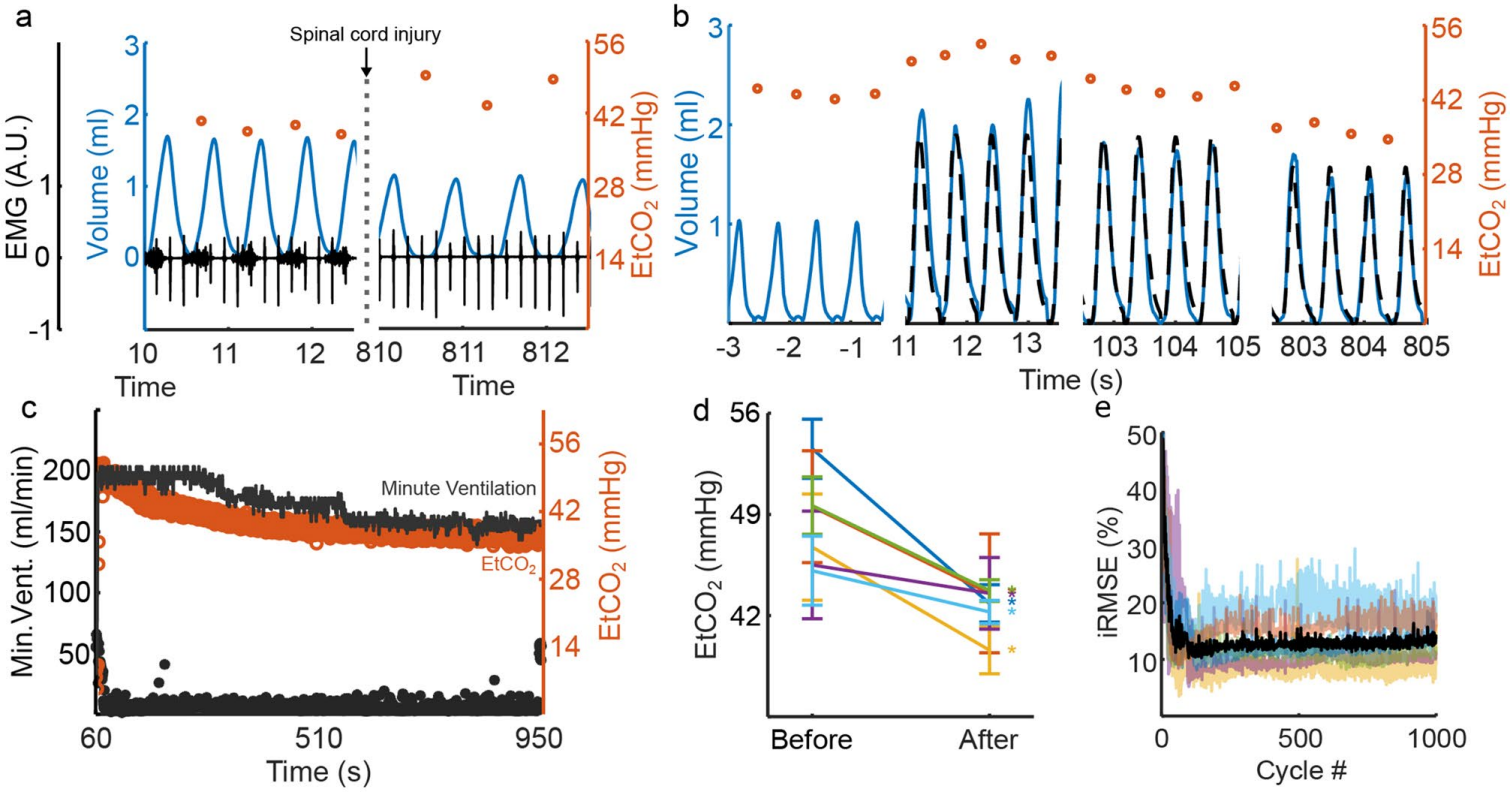
Adaptive PG/PS use in vivo after hypoventilation following C2 spinal cord hemisection. (**a**) Breath volume (blue), peak etCO_2_ (empty circles), and diaphragm EMG (black) in rats with intact spinal cord (left) and after C2 spinal cord hemisection (right). Hemisection of the spinal cord at the C2 level leads to paralysis of the hemidiaphragm ipsilateral to the injury as seen by a lack of bursts of EMG activity following SCI. This leads to hypoventilation and consequently, to an increase in etCO_2_. Black spikes in the EMG trace reflect artifacts from cardiac activity. (**b**) After initiation, the adaptive PG/PS controller generates a desired ventilatory pattern (black, dashed line) with elevated breath volume and shorter breath duration due to elevated etCO_2_. The PS then attempts to match the measured volume (blue, solid line) to the desired ventilatory pattern. At 100 s, the PS was able to match the measured breath profile to the desired breath profile, consequently causing a decrease in the etCO_2_ (orange, empty circles). By the end of the trial at 800 s, etCO_2_ is within a normocapnic range. Simultaneously, the desired tidal volume and respiratory rate generated by the computational model have decreased and the PS has adapted to match the measured tidal volume profile and respiratory rate to these. (**c**) Throughout the trial, the PG/PS controller is able to modulate stimulation parameters, as shown by the low iRMSE (black, solid circle), to elicit sufficient minute ventilation (grey line) to reduce etCO_2_ (orange, empty circles) to normocapnic values. After etCO_2_ is within the normocapnic range, ventilation is reduced and normocapnia is maintained for the rest of the trial. (**d**) The adaptive PG/PS controller was able to restore ventilatory function and reduce etCO_2_ significantly across all C2 spinal cord hemisected animals while (**e**) maintaining low iRMSE across all animals. Black line denotes average iRMSE across all animals.

In animals with SCI-induced hypoventilation, the PG/PS-controlled pacing achieved normocapnia by prescribing and generating an appropriate ventilatory pattern. A comparison of the ventilatory pattern and ipsilateral diaphragm electromyogram (EMG) before and after SCI is shown in Fig. 6a. Figure 6b shows how the PG/PS controller responded at the start of the trial, shortly after entrainment onset, and at the end of the trial. After initiation, the adaptive PG/PS controller elicited tidal volumes similar to or larger than those observed prior to injury. This increased tidal volume, coupled with decreased respiratory cycle duration led to an increase in minute ventilation and a decrease in etCO_2_. Once etCO_2_ decreased, the PG/PS controller updated the desired ventilatory pattern to maintain normocapnia. Figure 6c shows that the controller continuously modulated minute ventilation throughout the trial in response to etCO_2_ values and elicited breaths that matched the desired profile as shown by the low average iRMSE (9.1 ± 3.4%).

Across all SCI animals (Fig. 6d), the PG/PS controller was able to reduce etCO_2_ after onset of pacing and prior to the end of the trial from an average of 50.6 ± 5.7 mmHg to 43.1 ± 2.9 mmHg (*p* = 0.0064). The low average iRMSE (12.35 ± 1.97%) shown in Fig. 6e after entrainment in the SCI animals shows that the controller matched the inspiratory volume profile to the desired profile. This demonstrates that the adaptive controller is able to both determine and provide adequate respiratory pacing when endogenous activation of the diaphragm is impaired after SCI.

## Discussion

Diaphragm pacing approaches use manually selected “fixed settings”; there are no clinically available closed-loop respiratory pacing paradigms that can specify stimulation patterns in an automated fashion and adapt them to meet changing metabolic demands. The adaptive PG/PS closed-loop controller was designed to respond to abnormal etCO_2_ by adaptively modulating diaphragm muscle activation such that normocapnia can be achieved and maintained.

A computational testbed was used to select controller parameters and to test the ability of the PG/PS controller to respond to dynamic alterations in PaCO_2_ prior to experimental in vivo assessment. In simulations of changes in PaCO_2_ levels, the PG/PS controller with adaptation enabled was able to maintain arterial normocapnia over a wider range of metabolic demand than with adaptation disabled, thus demonstrating the value of modulating the ventilatory pattern.

The use of computational testbeds for development and testing of closed-loop neurotechnologies has seen rapid growth as it allows for relatively rapid testing and subsequent deployment of control algorithms prior to in vivo studies in a controlled and predictable environment and they are now being considered as a valuable regulatory tool^34^. In the present study, a computational testbed was particularly useful for the development of the chemoreceptor response algorithm. By simulating the effects of a sudden change in CO_2_ production and apneic events, it was possible to minimize their effect on controller stability by introducing an EMA with an appropriate time constant.

A computational approach could also facilitate future exploration and development of features not included in the current version, such as stimulation of abdominal muscles to effect active expiration. This could be implemented using an rCPG model that includes active expiration, such as that proposed by Molkov et al.^29^ and an additional PS module dedicated to control of abdominal musculature for use in an agonist/antagonist manner; as has been done previously for lower limb control^25,35^. A computational approach would speed up the development process and serve as a viable testbed for such additional functions.

In in vivo experiments, hypoventilation was induced via anesthesia or SCI. The adaptive closed-loop controller was able to increase ventilation and decrease etCO_2_ in both experimental groups via diaphragmatic pacing. This was successfully accomplished, without user intervention, despite hypoventilation through two different mechanisms, one in which respiratory rate had the largest influence in the reduction of minute ventilation (isoflurane-induced), and another in which reduced tidal volume was the largest factor (SCI-induced). In the former, the adaptive controller was able to cause entrainment of the intrinsic respiratory drive and elevate respiratory rate to mimic an adequate hypercapnic response by adjusting the respiratory cycle duration. Respiratory entrainment to the adaptive controller is likely to have occurred by activating pulmonary stretch receptors and engaging the Hering–Breuer reflex, as has been observed previously^18^, though it is also possible that intact afferent pathways may have affected intrinsic respiratory drive^36–38^. In the SCI group, the controller responded to the disruption of ipsilateral respiratory drive by adaptively modulating stimulation to bilaterally control the hemiparetic diaphragm to adjust breath volume.

To our knowledge, the technology does not currently exist to sample and measure PaCO_2_ in real-time and thereby enable arterial blood gas data to drive the PG/PS controller. However, while it is not an ideal replacement, etCO_2_ may be used to approximate PaCO_2_ under certain conditions^39^ and is commonly measured in clinical settings to guide medical decisions using standard technology^40^. However, under certain clinical conditions, the correlation between etCO_2_ and PaCO_2_ declines^39–42^. Usage of the adaptive controller under such circumstances would need to be more closely monitored and assessed to ensure adequate ventilation is being delivered. Therefore, etCO_2_ may be used as a practical and effective solution for clinical implementation of the PG/PS controller until further advances in PaCO_2_ measuring technology are available. Other considerations for clinical deployment are the effect of stimulation-induced diaphragm fatigue or compromised efficacy of complementary inspiratory muscles, such as after SCI. Co-stimulation of the intercostal muscles with the diaphragm could more effectively elicit the higher tidal volumes prescribed by the PG during elevated hypercapnia^43,44^ and reduce the work required from the diaphragm, thereby mitigating diaphragm muscle fatigue.

The ability to restore appropriate ventilation under two different mechanisms (respiratory depression and SCI) highlights the versatility of this adaptive PG/PS closed-loop approach and its potential for applicability to the clinical setting where hypoventilation may result from a variety of ventilatory impairments. These data further support the potential use of this controller in respiratory pacing applications to ensure adequate ventilatory function in response to dynamic changes in metabolic demand and/or lung/chest wall properties. However, additional studies are required prior to clinical implementation. These include evaluation of long-term closed-loop pacing efficacy, adaptability, and robustness; and addition of features to improve safety and function, such as reducing incidences of patient-pacing dyssynchrony.

The PG/PS controller could also be used under other circumstances that require respiratory assistance or when weaning from mechanical ventilation. In patients with Acute Respiratory Distress Syndrome (ARDS), as seen under some presentations of COVID-19, lung compliance decreases due to fibrosis after alveolar-capillary damage^45^. In such cases, adaptive pacing would be able to deliver stimulation to assist the patient’s intrinsic breathing and ensure that normocapnia is maintained regardless of changes in lung biomechanics. The PG/PS controller and temporary phrenic stimulation technology^46^ could be integrated with an oxygen supply with adequate positive end expiratory pressure (to minimize alveolar collapse). This integrated approach may allow patients to breathe in an assisted-as-needed manner, avoid or reduce volutrauma/barotrauma and diaphragm atrophy, decrease or avoid the weaning period, and require less supervision by trained professionals.

Here we have developed and tested a closed-loop adaptive controller with a neuromorphic architecture that has the potential for clinical impact on individuals who require respiratory assistance by providing automated real-time selection of stimulation parameter settings to achieve suitable ventilation in response to changes in metabolic demand. Since respiratory pacing is a life-sustaining technology, clinical deployment is contingent upon a robust demonstration that safety requirements can be met. If this is achieved, this controller could allow the user to engage in activities that elevate metabolic demand without risking hypoventilation. Indeed, even routine tasks that produce a change in posture (supine to sitting to standing) can evoke a significant increase in metabolic rate. An adaptive pacing controller would also prevent hyperventilation when metabolism drops, such as during sleep. Thus, our demonstration serves as an important step in the development of clinically feasible bioelectronic technology for use in acute or chronic respiratory insufficiency.

## Supplementary Information

Supplementary Information.

## Data Availability

The datasets generated during and/or analyzed during the current study are available from the corresponding author on reasonable request.
