# Supplementary material for: Autonomous control of ventilation through closed-loop adaptive respiratory pacing
Source: Sci Rep. 2020 Dec 14;10:21903. doi: 10.1038/s41598-020-78834-w (PMC7736353; doi:10.1038/s41598-020-78834-w)
Supplement: Supplementary file 1 — Supplementary Information. [file 41598_2020_78834_MOESM1_ESM.pdf]

Autonomous control of ventilation through closed-loop adaptive respiratory pacing

Authors: Ricardo Siu [1], James J. Abbas [2], David D. Fuller [3], Jefferson Gomes [1], Sylvie Renaud [4], Ranu Jung [1]*

Affiliations:

[1] Department of Biomedical Engineering, Florida International University, Miami, FL, USA

[2] School of Biological and Health Systems Engineering, Arizona State University, Tempe, AZ, USA

[3] Center for Respiratory Research and Rehabilitation, Department of Physical Therapy, University of Florida, Gainesville, FL, USA

[4] Université de Bordeaux, INP Bordeaux, IMS CNRS UMR 5218, Bordeaux, 33000, France

* 10555 W. Flagler St, EC 2602, Miami, FL, 33174, USA. Tel; 305-348-3722; Fax: 305-348-6954; email: rjung@fiu.edu

Supplementary materials and methods

PG/PS Controller design

The PG/PS controller design is based on the biological ventilatory control scheme, where the PG generates a ventilatory pattern and the PS adapts stimulation parameters to evoke a prescribed ventilatory pattern. The PG module includes a triphasic oscillatory network developed by Botros and Bruce to mimic the behavior of the respiratory CPG[26]. This rCPG model uses a CO_2-based input to determine an appropriate inspiratory duration which is then converted into a breath volume profile through the use of a chest biomechanical model. The volume profile and cycle duration are scaled and passed on to the PS to serve as the prescribed ventilatory pattern.

The rCPG network model used is composed of five interconnected neuronal populations. The activity of each of these populations is maximal at different phases of the respiratory cycle, these being early-inspiratory, inspiratory, late-inspiratory, post-inspiratory, and expiratory. These populations receive mostly inhibitory input from each other as well as from other sources, such as chemoreceptors and pulmonary stretch receptors. The equations for this oscillating respiratory network, as described in an earlier study[26], are,

$$\frac{dI}{dt} = -a_I I - W_{EI}S(E) - W_{PI}S(P) - W_{LI}S(L) + W_{II}S(I) + n_{CO_2}B_I \quad \text{(S1)}$$

$$\frac{dL}{dt} = -a_L L + W_{IL}S(I) - W_{EL}S(E) - W_{RL}S(R) + W_{LL}S(L) + B_L + W_{vL}v \quad \text{(S2)}$$

$$\frac{dP}{dt} = -a_P P - W_{EP}S(E) - W_{RP}S(R) + W_{PP}S(P) + n_{CO_2}B_P \quad \text{(S3)}$$

$$\frac{dE}{dt} = -a_E E - W_{IE}S(I) - W_{RE}S(R) - W_{EE}S(E) + n_{co2}B_E \quad \text{(S4)}$$

$$\frac{dR}{dt} = -a_R R - W_{IR}S(I) - W_{PR}S(P) + W_{RR}S(R) + n_{CO_2}B_R + W_{vR}v \quad \text{(S5)}$$

Where I, L, P, E, and R represent the inspiratory, late-inspiratory, post-inspiratory, expiratory, and early inspiratory neuronal populations, respectively, while v represents the input from the vagus nerve which carries pulmonary stretch receptor information. The self-decay term denoting the rate of decay is defined by a_i, where I is the respective neuronal population. This term allows for silencing of the i-population when no input is present. W_{ji} refers to the gain of the signal, or weight, of the j neuron to the i neuron, whereas W_{ii} is the self-activation factor. B_i refers to the weight of the chemoreceptor signal, n_{CO2}, which is considered to change linearly with $PaCO_2$. To convert firing frequency of the neuronal population to population activity, a sigmoid function, S, is used:

$$S(X) = K \cdot \alpha + (1 - K) \cdot \beta \quad \text{(S6)}$$

$$\alpha(X) = \begin{cases} \min(X, 4) & \geq 0 \\ 0 & < 0 \end{cases} \quad \text{(S7)}$$

$$\beta(X) = \frac{4}{1 + e^{-1.75 \cdot (X-2)}} \quad \text{(S8)}$$

Where X is the firing frequency and K is a constant that determines the steepness of the function.

The chemoreceptor input, n_{CO2}, is given as a bounded linear function of $PaCO_2$:

$$n_{CO_2} = \begin{cases} P_aCO_2 > 35 & n_{CO_2} = 0.2 \\ 45 < P_aCO_2 < 35 & n_{CO_2} = (PaCO_2 \cdot 0.072) - 2.32 \\ P_aCO_2 < 45 & n_{CO_2} = 1.72 \end{cases} \quad \text{(S9)}$$

The limits of 0.2 and 1.72 were set as a result of a set of preliminary simulations performed on the rCPG model to obtain the minimum and maximum n_{CO_2} values at which a physiologically relevant response was obtained. The linear function constants were set so that a linear response between a $PaCO_2$ of 35 to 45 mmHg is maintained.

To reduce the effect of transient events, an exponential moving average (EMA) of the peak $PaCO_2$ with a time constant, τ, of 8 sec was utilized. This was determined through simulations described in the main text.

To represent the pulmonary stretch receptor input, v, which contributes to the oscillatory behavior of the rCPG, a basic model to represent pulmonary expansion[26] is used,

$$\frac{dv}{dt} = -K_1 \cdot v(t) + K_2 \cdot S(I) \tag{S10}$$

Where $K1$ and $K2$ are constants set such that v increases during inspiration but decays as $v(t)$ increases, reflecting the Hering-Breuer reflex. When this model is paired with the previously described respiratory CPG equations and chemoreceptor model, rhythmogenesis occurred, producing a ventilatory response to $PaCO_2$ similar to that observed in mammals[26]. Given that this basic model creates a pattern that represents pulmonary stretch, it can be used to derive a breath volume profile.

The output of the inspiratory pool of the rCPG was half-wave rectified and processed through the pulmonary stretch receptor model. The pulmonary stretch receptor output was then scaled in amplitude to match the tidal volume expected for the weight of each rat[27]. The range of the cycle duration of the rCPG output was also scaled in time to match the range of breath durations observed in rats under eupneic[27,28] to hypercapnic[28,29] conditions. This scaled ventilatory pattern then provided, on a breath-by-breath basis, a prescribed trajectory for the PS module to follow. In the experimental studies, if $etCO_2$ information is unavailable (e.g. first breath of pacing), the controller worked under the assumption that the $etCO_2$ input is 36 mmHg.

The PS module for respiratory control has been described in a previous study[18]. The PS module aims to determine adequate stimulation parameters to elicit a specified breath volume profile. In

previous studies, this prescribed profile was preset based on baseline breath volumes and profiles for each rat; here, the prescribed profile was generated by the PG module. Briefly, the PS consists of a single-layered neural network with time-shifted activation profiles. The output of the controller, z, is a value from 0 to 1 which is multiplied by the maximum allowed current amplitude. This is given by

$$z(t) = \sum_{j=1}^{n_a} w_j(t) y_j(t) \tag{S11}$$

The output is defined by the summation of the weighted output (y_j) of all active neurons, n_a. Neuronal weights for each neuron j are given by w_j.

The PS uses a comparator to define the error at any time t, $e(t)$, between the prescribed volume trajectory defined by the PG module and the measured volume profile. The change in weight, Δw_j, for all neurons at time t is defined by

$$\Delta w_j(t) = \eta e(t) \sum_{k_p=1}^{n_p} \frac{1}{n_p} y_j\left(t - k_p T\right) \tag{S12}$$

Where η is the learning rate, n_p is the number of past activations over which the error is time averaged to account for delays in activation, and $y_j(t-k_pT)$ is the output of neuron j at previous times. Hence, the error at time t affects all neurons that have been recently active; the amount of change is proportional to its activity over the specified window (n_pT).

The neural network contains a maximum of 72 neurons time shifted every 0.014 sec to span a duration of 1.05 seconds. To account for changes in breath cycle duration, the network is re-organized by excluding a certain number of neurons of the network at the start of every breath to match the new prescribed cycle duration. Thus, as $PaCO_2$ increases and the PG module prescribes a ventilatory pattern with reduced cycle duration, neurons with zeroed weights (an indication of

no influence on stimulation) are excluded. This shortens the cycle such that the updated cycle duration closely matches that of the shortened prescribed breath duration. Once $PaCO_2$ decreases and the PG module prescribes an increase in breath cycle duration, these neurons are included to prolong the cycle duration. In this manner, the PS is able to work in concert with the PG module to evoke the prescribed ventilatory pattern.

The PG/PS controller was programmed and implemented in LabVIEW (National Instruments, Austin, TX) for both *in vivo* and *in silico* studies. All controller constants can be found in Supplementary Table S1. In animal studies, the controller output to the stimulator was scaled such that the maximum allowed current amplitude was four times the twitch threshold, as previously described[18].

Table S1. Controller parameters and computational model constants

r	1.1	W_{EI}	1.371	W_{PI}	1.719	W_{LI}	5.0	W_{II}	0.7
W_{IL}	1.361	W_{EL}	0.793	W_{RL}	2.056	W_{LL}	2.3	W_{vL}	10.5
W_{EP}	1.351	W_{RP}	2.254	W_{PP}	1.540	W_{IE}	0.729	W_{RE}	2.254
W_{EE}	1.550	W_{IR}	1.80	W_{PR}	2.150	W_{RR}	0.650	W_{vR}	1.05
K	0.6	k_d	7 gcm^3/s^2	K_1	2.5	K_2	4	m_L	1.8 g
B_L	8.4 g/s	K_L	11.5 g/s^2	n_a	17	n_p	6	η	0.001
T	0.014 s	V_T	297.72 ml	V_B	2.28 ml	M_T	0.49	Q	1.2875 ml/s
Q_T	1.2512 ml/s	Q_B	0.0363 ml/s	C_{in}	0.01	τ_{AT}	0.027 s	τ_{AB}	0.01 s
τ_{VT}	0.1 s	τ_{VB}	0.02 s						
